# Foreign Bodies in the Air Passages

**Published:** 1897-10-02

**Authors:** 


### FOREIGN BODIES IN THE AIR PASSAGES.

A couple of cases recently reported serve to draw attention afresh to the important *rôle* which foreign bodies in the air passages often play in the production of disease of the respiratory organs.

One is a case[1] in which a pipe-stem is stated to have remained impacted in one of the bronchi for a period of three months. The patient had a faint in the street and fell. On recovering he found that his pipe was broken, and the mouth-piece end, about an inch in length, could not be found. After this he complained of a pain more or less severe in the left breast, with dyspnœa on exertion, and a profuse muco-purulent expectoration. No physical signs of obstruction to the access of air to any portion of the lung were discovered. Three months afterwards, however, while carrying a heavy weight in a stooping posture, he felt something move in his chest. This was followed by a fit of coughing, and the long-lost pipe-shank dropped out of his mouth on to the ground, surrounded by a little coagulated blood and mucus. He immediately felt relief to the embarrassment in his breathing.

The other case recorded by Dr. Bunch and Mr. Lake[2] is much more fully reported, and is of considerable interest, not only from the length of time during which the foreign body seems to have remained in the bronchus and the fact that it ultimately became loosened and put the patient in serious danger of immediate suffocation from its impaction in the trachea, where its irritation caused the growth of a mass of granulative tissue by which stenosis was produced, but from the fact that the nature of the disease was not revealed for a long time, even after the most careful examination. The case had, indeed, been exhibited at one of the laryngological societies as one of tracheal stenosis of specific origin. There was a history of a bone having "stuck in her throat," and caused a violent attack of suffocative dyspnœa nearly nine years before, followed by a soreness of the throat, which continued more or less for a month. For three or four months after this the breath seems to have been good, but then the patient had a cough which lasted all the winter. This was repeated every winter, with some pain at the base of the right lung; but this was all she complained of until in July, 1896, she was suddenly attacked with a violent cough, with some hæmoptysis, followed by huskiness of voice and soreness in his throat, especially in swallowing. After this the breathing became progressively worse. There was evidently some tracheal stenosis, paroxysms of dyspnœa came on, and on December 18th Mr. Lake discovered a foreign body lying across the trachea, the ends being buried in an ulcerated inflammatory swelling which caused considerable narrowing of the trachea. On January 25th the trachea was opened by Mr. Watson Cheyne, and the foreign body, a thin shell of bone, was removed, after which the patient rapidly recovered.

For many interesting particulars in regard to the effects produced by the retention of foreign bodies for lengthened periods in the bronchial tubes, reference may be made to a paper by Mr. Godlee,[3] read before the Royal Medical and Chirurgical Society on March 24th, 1896, and published in the "Transactions."

In this paper a case is given in which an abscess in the lung had been caused by the presence of a bit of rabbit bone. The abscess was opened, but no foreign body was found. Ten months afterwards, however, the piece of bone was coughed up. Those interested in such matters may usefully compare with this a case of abscess of the lung due to localised necrosis, reported last week by Dr. Mitchell Clarke and Mr. Charles Morton,[4] occurring in a man subject to epilepsy, and attributed to the inhalation of sewer gas. The possibility of the necrosis having been set up by a foreign body was borne in mind, but nothing of the sort was found, either at the time of operation or at the necropsy. Still, the whole history has a curious similarity to cases caused by foreign bodies, and one must not forget the possibility of such a thing being ejected during one or other of the violent paroxysms, which, it is said, made the patient "black in the face."

[1] Brit. Med. Journ., Sept. 25, 1897. [2] Lancet, Sept. 25, 1897. [3] Trans. Roy. Med. Chirurg. Soc., vol. lxxix. [4] Lancet, Sept. 25, 1897.

